# The mechanical and photoelastic properties of 3D printable stress-visualized materials

**DOI:** 10.1038/s41598-017-11433-4

**Published:** 2017-09-07

**Authors:** Li Wang, Yang Ju, Heping Xie, Guowei Ma, Lingtao Mao, Kexin He

**Affiliations:** 1 School of Mechanics and Civil Engineering, China University of Mining & Technology at Beijing, D11 Xueyuan RD, Beijing, 100083 China; 2 State Key Laboratory of Coal Resources and Safe Mining, China University of Mining and Technology at Beijing, D11 Xueyuan RD, Beijing, 100083 China; 3 State Key Laboratory for Geomechanics and Deep Underground Engineering, China University of Mining and Technology at Xuzhou, 1 University Ave, Xuzhou, 221006 China; 4 Key Laboratory of Energy Engineering Safety and Mechanics on Disasters, The Ministry of Education, Sichuan University, Chengdu, 610065 China; 5 School of Civil, Environmental and Mining Engineering, The University of Western Australia, Crawley, WA 6009 Australia

## Abstract

Three-dimensional (3D) printing technology integrating frozen stress techniques has created a novel way to directly represent and characterize 3D interior discontinuities and the full-field stress induced by mining- or construction-related disturbances of deeply buried rock masses. However, concerns have been raised about the similitude between the mechanical behaviours of the printed model and its prototype rock mass. Ensuring the mechanical properties of the printable materials are as close as possible to those of real rock mass is of critical significance. In this work, a transparent, light, photosensitive polymer material was investigated for applications in frozen stress tests. The chemical composition of the material was determined by integrating the results of infrared spectroscopy (IR spectroscopy), X-ray diffraction (XRD), pyrolysis, gas chromatography and mass spectrometry (PY-GC/MS). Measures to improve the mechanical properties of the printable material, including printing orientation, post-processing, and temperature control, were evaluated by comparing the treated material with its prototype rock. The optical stress sensitivity of the material, including stress-visualized properties and stress-frozen performance, was also tested. This study offers an understanding of how printable materials should be modified to better simulate real rock masses, in terms of not only their geological geometry but also their mechanical performance.

## Introduction

Mining and construction activities on deeply buried rock masses may induce large deformations in the surrounding rocks and disturbances in the geo-stress field, resulting in serious geological hazards^1–4^. An accurate characterization of the complex stratum structure and stress field is essential to optimize the exploitation of energy and other resources stored deep underground and for effective early warning, prevention and control of rock disasters caused by such exploitation. However, the invisible and intangible nature and complex structure of rock matrices and diverse geological bodies make it extremely difficult to directly display the complex stratum structure and physically extract the stress field. These issues have become a bottleneck in traditional detection methods and theoretical analysis for characterizing the 3D discontinuities and full stress fields of rock masses.

3D printing technology integrated with frozen stress techniques offer a novel, promising way to physically display complex underground structures and to probe strata stress fields^5–7^. 3D printing has been widely applied in the fabrication of various 3D physical objects with complex shapes^8, 9^. It has been proved to show a great potentiality when applied to solve some rock mechanics problems^10–13^. However, these reported works were not capable of physically and directly presenting the stress field and its evolution inside media, which is a key factor for solving relevant rock mechanic problems. The advanced commercially available 3D printer Objet Connex series uses PolyJet 3D printing technology and possesses the unique capability of printing the highest number of different materials reported thus far^14–16^. VeroClear is one of the printable materials that can be used in Objet 3D printers. It is transparent and possesses favourable stress-optics sensitivities. This material has been successfully employed to produce a physical model that represents natural rock^17^. The ultimate goal of our study is to provide the applicability of 3D printing and photoelastic techniques to the quantitative visualisation and characterization of the stress fields of practical rock mass with heterogeneous structures. Preliminary researches were conducted^5–7^ and have attracted attention^10, 18–20^. However, a certain mismatch exists between the printed model and its prototype rock mass. This mismatch is related to the mechanical and deformation performance due to the intrinsic nature of these materials. To ensure that the physical and mechanical response of deep rock masses can be accurately reflected by 3D-printed geological models, improving the similitude between the physical and mechanical behaviours of the printed model and real underground rock is of critical significance.

However, due to the different sedimentary and forming processes of natural reservoir rocks, their interior structure and physical and mechanical properties vary widely, which leads to great difficulties in developing 3D printing materials that are transparent, optical stress sensitive and that possess the same or similar mechanical properties as those of natural rocks. Printing models that are identical to real rock samples in terms of mechanical properties remains a significant challenge. The raw material and manufacturing process may be assumed to exert a large influence on the mechanical response of objects constructed by 3D printing, and a certain degree of anisotropy will be introduced in the mechanical properties of printed models due to their stacked-layer nature^14, 16^. Most published results indicate that the mechanical properties of PolyJet manufactured parts are dependent on ultraviolet light (UV) exposure time and building orientation^14, 21^. On the other hand, post-treatments using heat are widely used to alter the physical and chemical properties of a material and can sometimes be applied to achieve a specific desired result, such as hardening or softening of a material. There is a consensus in the literature that, in general, heat treatment can enhance the mechanical properties of cured composites^22–25^. Heat treatment may help relieve the processing stress originating during resin polymerization and finishing procedures^26^. Moreover, post-cure heating of resin materials can decrease the levels of unreacted monomer after the initial light-curing stage^27, 28^, and it can help improve stability and reduce the UV exposure time-dependent properties of the adopted material.

Several studies have investigated the mechanical properties of printing materials used in PolyJet 3D printers, considering the infill pattern, building orientation, layer thickness, heating treatment, etc.^10, 29–33^. However, most of the investigated printing materials are opaque and non-stress-visible (i.e. do not make stress visible). Previous test results cannot be directly applied to characterize the properties of VeroClear because the different printing materials result in different physical and mechanical properties. Few published results have investigated the impact of post-heat treatment on the mechanical properties of 3D printing materials solidified by UV light curing. Additionally, to date, few studies are available analysing the chemical composition and characterizing the properties of this printing material.

The main objectives of this research are to introduce a transparent, photoelastic printable material, to study and modify its physical and mechanical properties, and to improve the similarity between its strength and stiffness and those of natural rocks. First, its principal chemical constitution is determined. Subsequently, the impacts of building orientation and heat-treatment temperatures on the mechanical properties of 3D-printed samples are evaluated, including on the uniaxial compressive strength, direct tensile strength, triaxial compressive strength etc. Lastly, its unique stress-visualized performance is investigated. This study is expected to provide a reference for the further modification of printable materials that can accurately simulate natural rock in terms of geometry and mechanical properties. Additionally, this study also aims to provide a way to uncover the intrinsic governing mechanisms of engineering-related geological disasters.

## Results

### Chemical composition analysis

**Figure 1:**
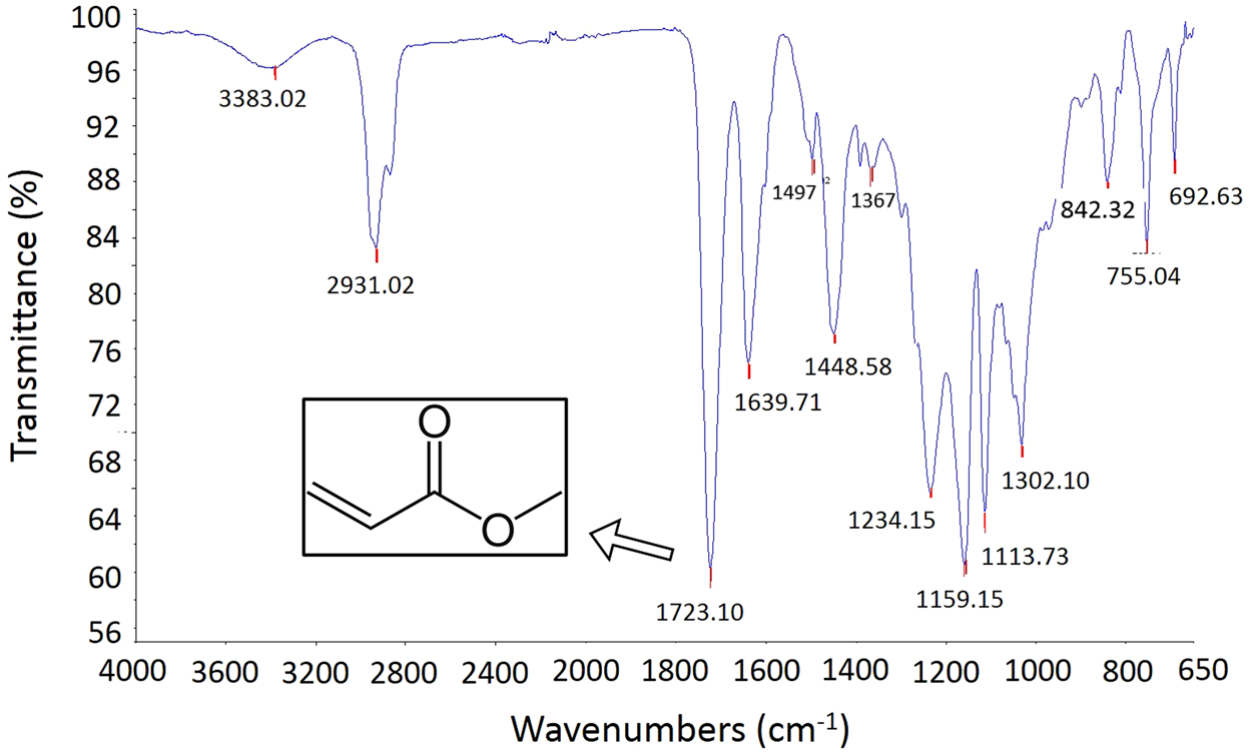
Infrared spectrum of VeroClear.

**Figure 2:**
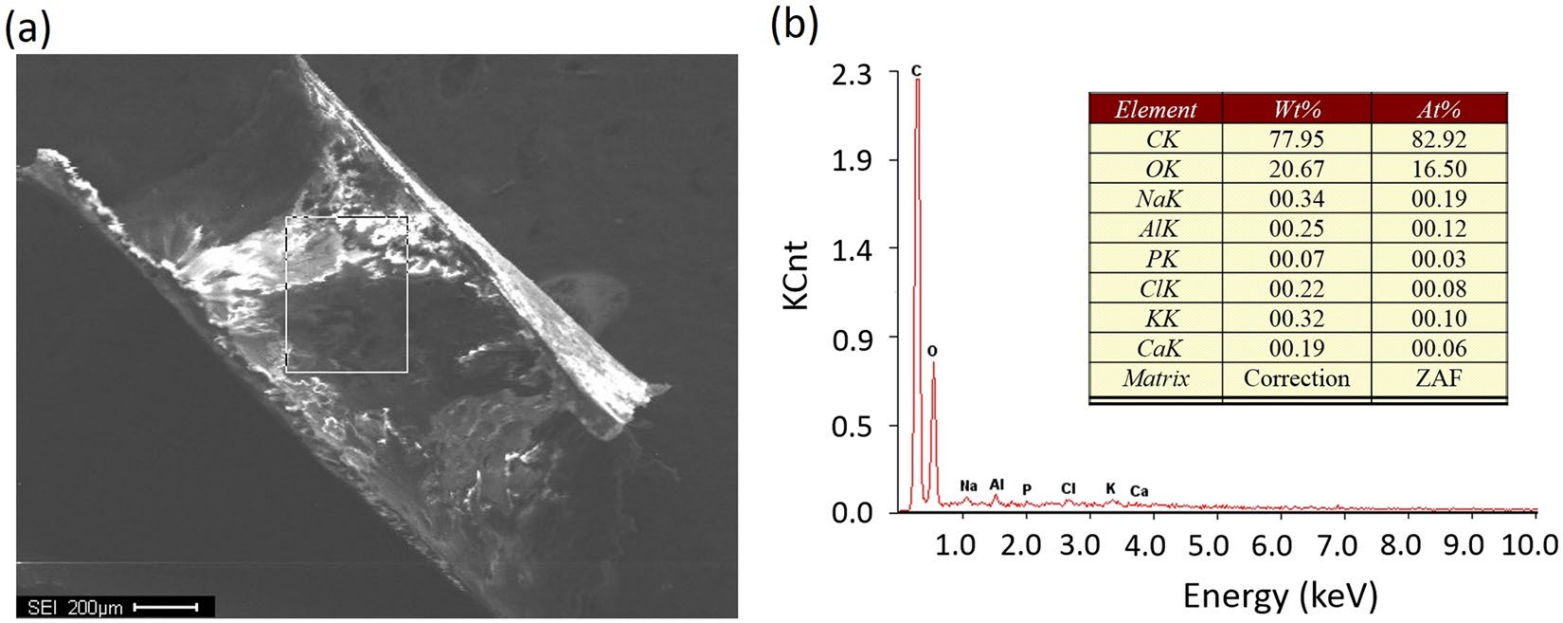
**(a)** SEM image of UV-cured VeroClear; **(b)** XRD diffractogram and an analysis of its results (inset).

**Figure 3:**
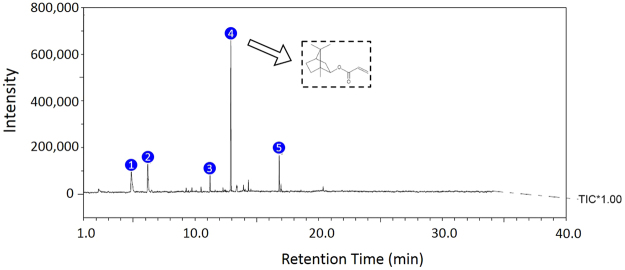
Total ion chromatogram obtained from the pyrolysis of VeroClear at 300 °C.

Figure 1 shows the IR spectra of VeroClear, wherein the solid line traces the values of transmittance for every wavenumber. The peak at a wavenumber of 1723.10 indicates the presence of acrylate (a family of polymers). The microstructure of UV-cured VeroClear was assessed using scanning electron microscopy (SEM), as illustrated in Fig. 2(a). Subsequently, the composition and content of VeroClear were quantitatively analysed using XRD patterns, as shown in Fig. 2(b). VeroClear primarily comprises two chemical elements, carbon and oxygen, which account for 82.92% and 16.50% of the composition, respectively. Figure 3 plots the recorded total ion current obtained from PY-GC/MS experiments. VeroClear is pyrolysed at 300 °C. Characterization of the pyrolysis products reveals that the major component is isobornyl-acrylate (IBOA), indicated by the fourth peak in the total ion chromatogram. The total percentage area of the IBOA is 51.2%. IBOA is characterized by low shrinkage and high hardness, and its molecular formula is C_13_H_20_O_2_.

### Effect of building orientation

**Figure 4:**
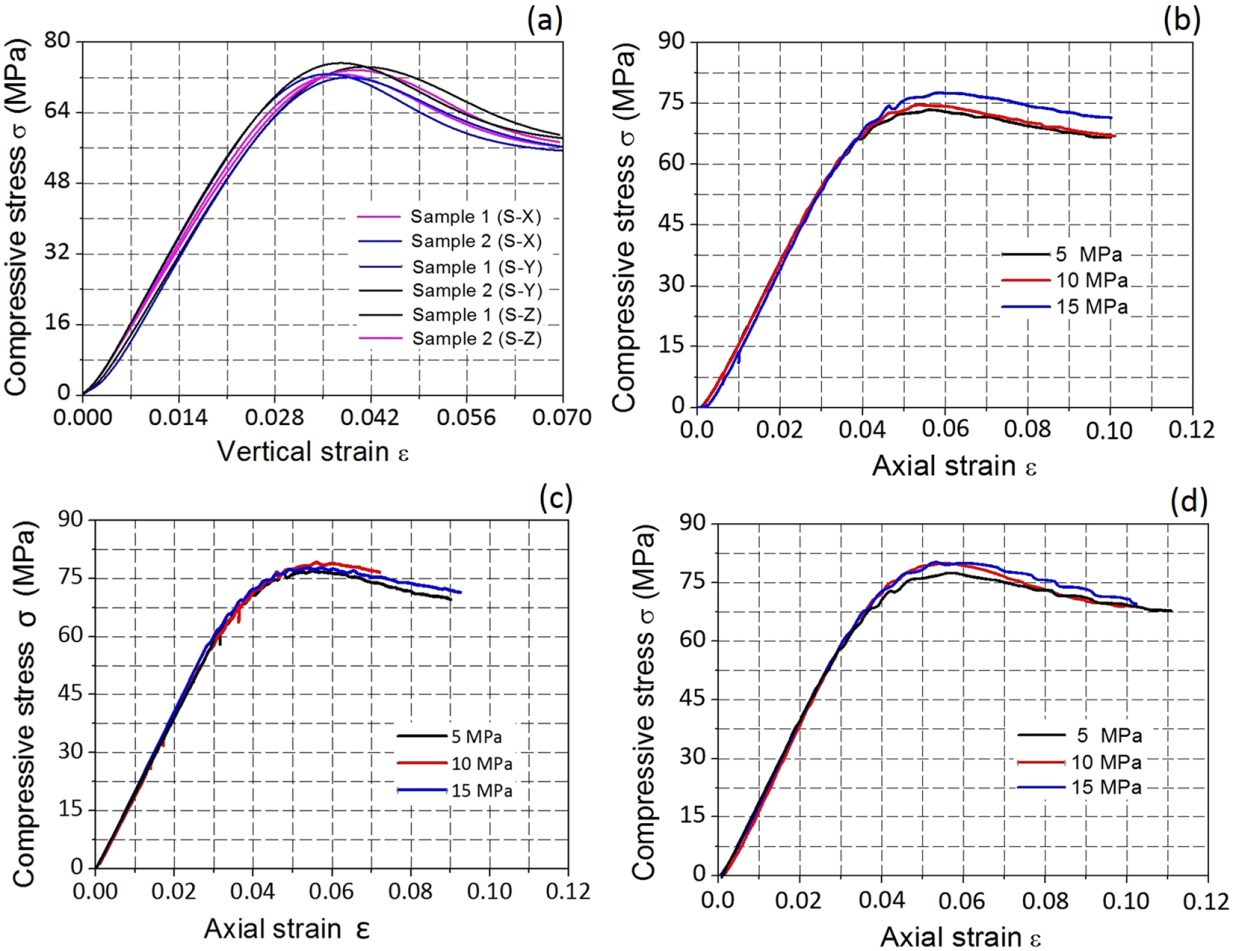
Stress–strain curves of 3D printed cylindrical samples built up by different methods under (**a**) uniaxial compressive conditions and (**b–d**) triaxial compressive conditions. Images **b**, **c** and **d** represent the building types S-X, ﻿S-Y and S-Z, respectively.

Figure 4(a) illustrates the stress–strain curves of the specimens printed using different methods under uniaxial conditions. The results indicate that all printed specimens deform like a ductile material. The average UCS of three samples of S-Z is 3.7% higher than that of S-X and 4.4% higher than that of S-Y. The Young’s moduli at 50% of the ultimate strength are calculated for each specimen from their relevant stress–strain curves. The average Young’s moduli (*E*) measured for the specimens S-X, S-Y and S-Z are 2.21, 2.20, and 2.29 GPa, respectively. Samples with horizontally printed layers (S-Z) exhibit values approximately 3.85% higher than those with vertical layers (S-X and S-Y). Figure 4(b–d) present the stress–strain curves of 3D-printed specimens under triaxial loading conditions. The deformation curves indicate that all the models deform like typical ductile materials under triaxial conditions, similarly to those exposed to uniaxial compression. The *f*_c_ values of the S-Z samples are 5.59%, 7.27% and 3.29% higher than those of the S-X samples when exposed to confining pressures of 5 MPa, 10 MPa and 15 MPa, respectively. Moreover, the *E* values of the S-Z samples are 7.2%, 7.8% and 14.4% higher than those of the S-X samples when exposed to confining pressures of 5 MPa, 10 MPa and 15 MPa, respectively. In general, under triaxial conditions, the samples with horizontal layers (S-Z) show higher strength and stiffness than those with vertical layers (S-X and S-Y). However, the experimental results showed little variance of triaxial compression strength under different confining pressures, which can possibly be attributed to the intrinsic physical nature of the polymer printing material being different from those of natural geomaterials.

### Effect of heat treatment

**Figure 5:**
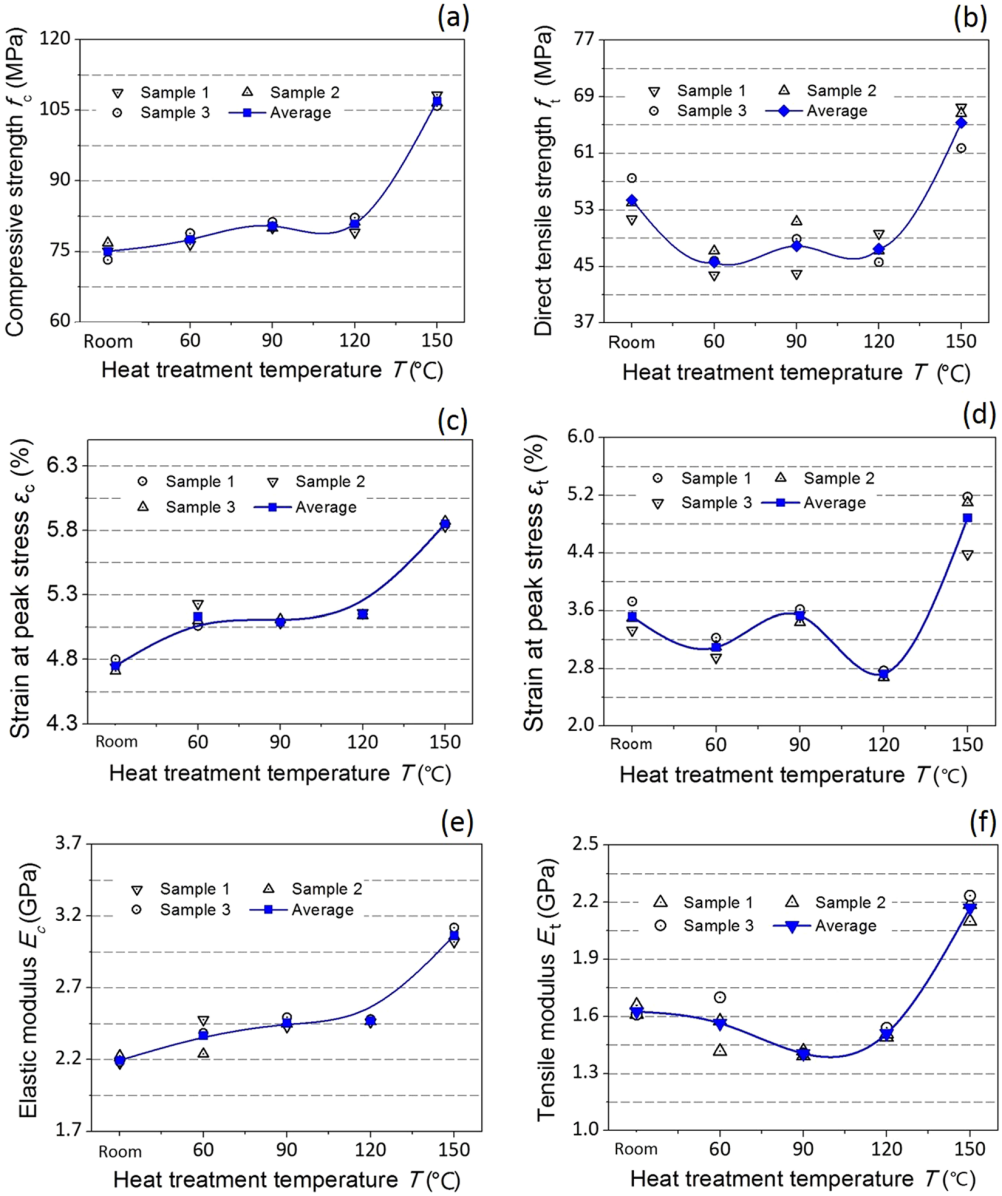
(**a**) Uniaxial compressive strength, (**b**) direct tensile strength, (**c**) strain at peak compressive stress, (**d**) strain at peak tensile stress, (**e**) Young’s modulus and (**f**) tensile modulus of printed specimens after heating post-treatment.

Figure 5 illustrates the uniaxial compressive strength (*f*_c_), strain at peak compressive stress (*ε*_*c*_) and Young’s modulus (*E*_*c*_) of printed specimens after post-treatments at different temperatures. The post-treatment can have a larger impact on the mechanical properties of 3D-printed models than the build-up orientations. Evidently, little difference exists between the three tested samples heat treated at the same temperature. As illustrated in Fig. 5, the mechanical properties of the specimens treated at 60 °C, 90 °C and 120°C are slightly higher than those of the untreated specimen. The highest UCS, 106.9 MPa, was obtained for the specimen treatment at 150 °C, which is 32.2% higher than that obtained with treatment at 120 °C and 48.7% higher than that of the untreated specimen. The measured strain at the peak compressive stress (*ε*_*c*_) and Young’s modulus (*E*_*c*_) for the specimens treated at 150 °C are 5.85% and 3.26 GPa, respectively. Heat treating at 150 °C increases the *ε*_*c*_ and *E*_*c*_ of the specimens by 23.2% and 48.7%, respectively, relative to those of untreated specimens. Increasing the temperature to 150 °C obviously affected the direct tensile strength of the 3D-printed specimens. The DTS, *ε*_*t*_ and *E*_*t*_ of the specimens after heating to 150 °C are 65.34 MPa, 4.89% and 2.17 GPa, respectively. Heat treating at 150 °C increases the DTS, *ε*_t_ and *E*_t_ of specimens by 20.11%, 38.83% and 33.46%, respectively, relative to those of untreated specimens. The mechanical properties significantly improve when the post-processing temperature is changed from 120 °C to 150 °C, as shown by increases in DTS, *ε*_t_ and *E*_t_ by up to 37.59%, 79.25% and 43.71%, respectively.

### Stress visualization performance

**Figure 6:**
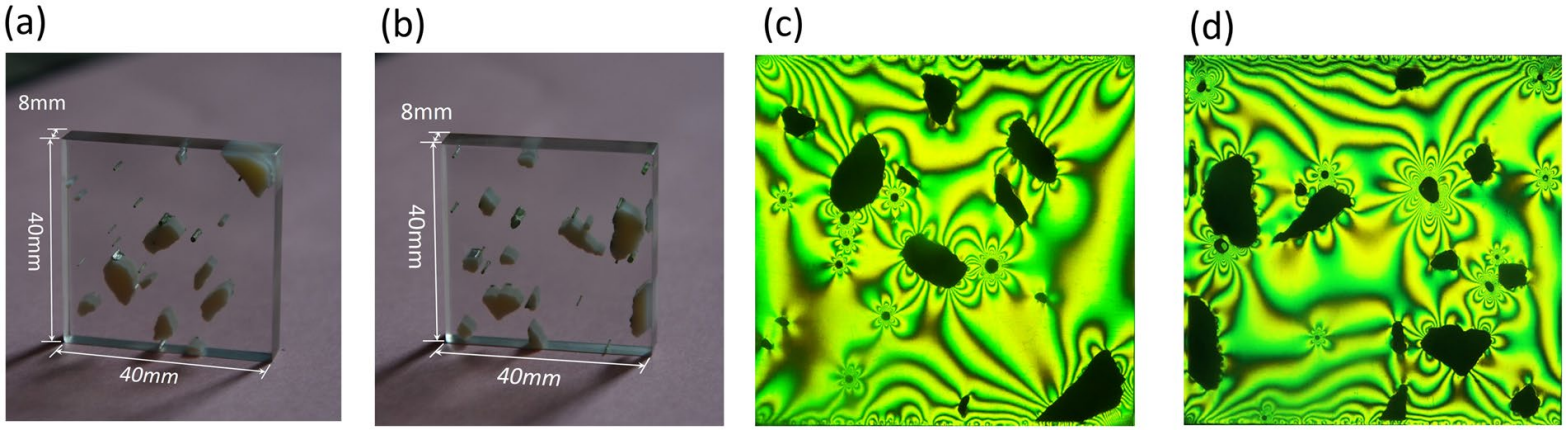
(a,b) 3D–printed aggregated models; **(c,d)** visualizations of stress fields obtained by the photoelastic method.

**Figure 7:**
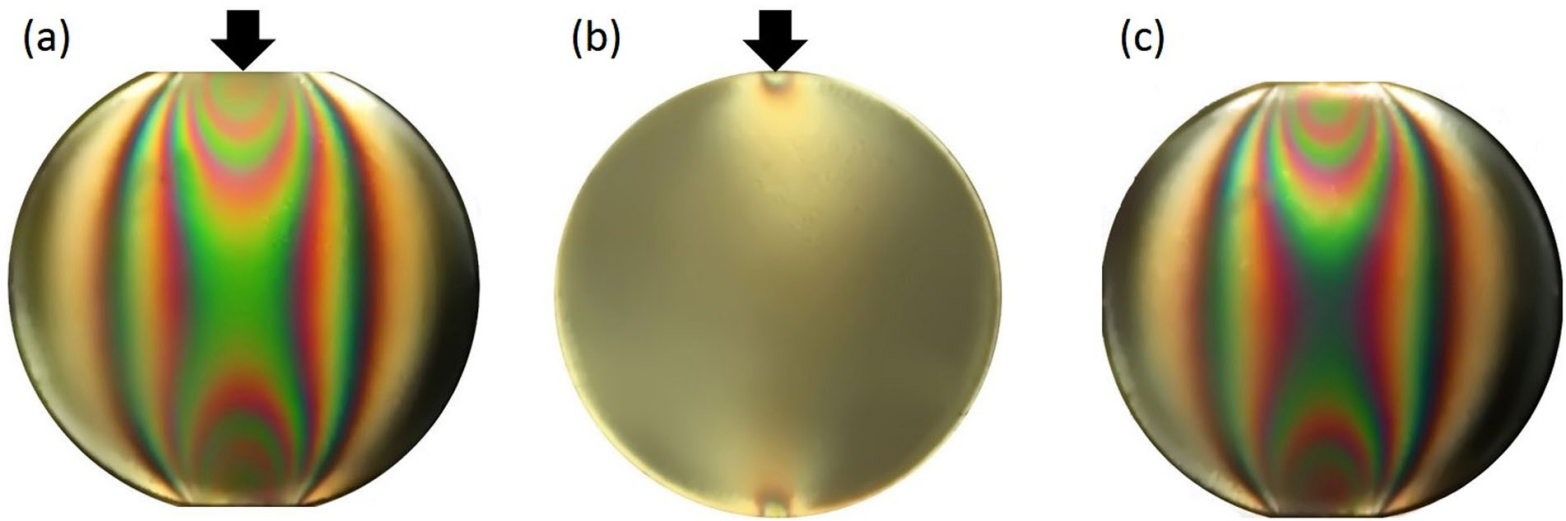
Colour fringe maps of a disc (**a**) at room temperature and under a pressure of 60 N, (**b**) at 60 °C and under a pressure of 60 N, and (**c**) after recovery to room temperature and without compression.

Figure 6(a,b) present two 3D-printed aggregated models made using the transparent material VeroClear as the matrix embedded with particles of the white, opaque printable material RGD. Figure 6(c,d) show visualizations of the stress field of aggregated models exposed to uniaxial compressive pressure, presented as monochromatic isochromatic images. Two models were selected to illustrate the feasibility and effectiveness of the proposed material and method to visualize the stress field of different structures. Figure 7 shows our preliminary exploration to assess the stress-freezing property of VeroClear at a temperature lower than its critical temperature of 120 °C. More information can be found in ref. 5, 7. The stress-frozen property is an inherent characteristic of photoelastic materials, which is independent of the geometry of the studied models. A circular disc diametric compression was selected to reach a consensus with the already reported investigations, and in particular to validate the visualisation method for the stress field by comparing the experimental results with the theoretical solutions. The disc exhibits a stress-freezing cycle and the coloured fringe figures were captured at specific stages. These observations indicate that VeroClear exhibits distinct stress-freezing at 60 °C, which is much lower than its critical freezing temperature.

## Discussion

In terms of the compositional analysis, the test result in Fig. 1 demonstrates that the printing material VeroClear is an acrylate-based polymer. Acrylate polymers are noted for their transparency, resistance to breakage, and elasticity^34^ and are promising for applications as materials for printing transparent, complex structures simulating heterogeneous rocks. Moreover, acrylate polymers also belong to one of the three classes of photo-curable resins^35, 36^. Figure 2 shows that the cured system is characterized by a type of amorphous, crosslinked structure. During printing, the photopolymer undergoes a process called photo-curing, wherein oligomers are crosslinked upon exposure to UV light. This curing results in the formation of a thermoset network of polymers that form into a hardened polymeric material.

In terms of the effect of build-up orientations on the mechanical behaviour of the printed samples, three possible factors are derived from the test data: the orientation of the layers, the UV exposure time and the support material. According to the PolyJet construction procedure, each sprayed layer must be cured before the next layer is printed on the previous one, resulting in a laminated structure. The vertical layers of S-X and S-Y are parallel to the applied load direction, while the horizontal layers of S-Z are perpendicular to the loading direction. Theoretically, building orientations have a certain effect on the mechanical behaviour of the printed samples. However, the utilized material, VeroClear, is a type of liquid polymer, and the printed-layer thickness of the printed samples is in the range of 16–30 μm, which is considerably thin. High-intensity UV radiation has a highly desirable capability to instantaneously harden the sprayed polymer. Therefore, the adopted material and modelling precision can both significantly reduce the effect of the layered structure on the macroscopic response of the printed samples. On the other hand, the printing time, i.e., the time for which the sample is exposed to UV light, may contribute to differences in the mechanical behaviour. The constructed height of the samples is proportional to the duration of UV light exposure. The printing time of S-Z (construction height 10 mm) is nearly twice as long as that of S-X and S-Y (construction height 5 mm). Previous studies have shown that an increase in the UV exposure time produces a hardening effect, in part produced by photo-polymerization^37^. Therefore, the UV exposure time is the main factor affecting the mechanical properties of the printed samples. Additionally, the support material may introduce a shielding effect against over-curing certain surfaces with UV light, thus reducing the strength and stiffness of the material^14^. Accordingly, a longer UV light exposure duration and a less support material can explain why the S-Z samples possess higher strength and stiffness than the S-X and S-Y samples.

We hold that the degree of influence that the manner of building has on the properties of a printed specimen depends on the type of materials used in the printer. PolyJet printing is a liquid-based process, which makes the printed specimens more homogenous than those fabricated by powder-based or filament-extrusion 3D printers. Test results indicate that the UCS and Young’s moduli of the specimens printed by a powder-based process can vary by up to 300% and 400%, respectively^10^. However, the differences in the UCS and Young’s moduli of specimens printed by a liquid-based process are within 18% and 8%, respectively^29^.

In terms of the effect of the heat-treatment temperature on the mechanical behaviour of printed samples, the mechanical properties of specimens constructed of VeroClear are significantly enhanced as the heat-treatment temperature is increased from 120 °C to 150 °C. Previous studies have reported that a heat treatment above the glass transition temperature (*T*_g_) can improve the mechanical properties of the treated polymeric materials^38, 39^ because the applied temperature results in an effective increase in polymer chain mobility during the polymerization process, which can lead to further monomer conversion and improvement in the crosslink density^26, 40^. The glass transition temperature of polyacrylate is predicted to be in the range of 82–105 °C^41^. Therefore, the *T*_g_ of the polymer VeroClear (acrylate) is very likely to be in the range of 120–150 °C. The hot-light curve of VeroClear, which characterizes the relationship of deformation and temperature, exhibits a critical temperature of 120 °C. At this temperature, the material undergoes a transformation from the glassy state to the rubbery state. This further demonstrates that the critical glass transition temperature (*T*_g_) is located at 120 °C. During the transition of a polymer from the glassy state to the rubbery state, molecular chains of the polymer become more mobile in response to the development of a cohesive, entangled network in the polymer. This contributes to structural compactness and therefore the development of high stiffness^42^. This result provides supplemental evidence for the large enhancement of the mechanical properties of VeroClear upon exposure to temperatures higher than 120 °C. However, an accurate identification of the critical glass transition temperature *T*_g_ of the polymer VeroClear must rely on further assessment by differential scanning calorimetry (DSC). This result will help explain the enhancement of the mechanical properties as the heat-treatment temperature is increased from 120 °C to 150 °C.

In terms of stress visualization, the printing material we used in this work not only exhibits good birefringence and photoelasticity at room temperature but can also show stress-freezing when exposed to a temperature higher than its critical freezing temperature (*T*_c_). Based on the above investigations, VeroClear clearly exhibits prominent optical stress sensitivity. In particular, this enables the isochromatic fringes around force-bearing points or discontinuities to be clearly displayed and captured. The presented results can prove the applicability of the printing material to visualize the stress field of the slices extracted from the heterogeneous model after the stress-frozen test. The stress-visible material has great potential in solving various engineering problems that are associated with the 3D full-field stress, such as unconventional oil and gas extraction, reinjection of hydraulic fracturing waste, CO_2_ geological sequestration, disposal of nuclear waste, seismic prediction, geothermal energy utilisation, civil and building construction, as well as deep underground coal mining^43–47^. 3D printing and photoelasticity have paved a novel and promising way to analyse the catastrophic problems encountered in various engineering activities.

In summary, adjusting the build-up direction and particularly post-processing the material above its glass transition temperature can increase the strength and stiffness of the printing material. Both measures contribute to improving the similitude between the printing material and natural rocks to a certain extent. However, natural rocks inevitably contain many initial random imperfections, such as micro/meso-scale pores, fractures and joints^48–50^. These physical characteristics considerably vary the mechanical behaviour of rocks. Therefore, simulating natural rocks with current photosensitive printing materials remains a significant challenge. This is particularly true with respect to the difference between the ductility of the printing material and that of natural rocks. Our on-going research efforts include investigating ways to improve its similitude to natural rocks. The measures include: (1) developing new printable materials, which keep both favourable transparent and stress-visible properties similar to VeroClear, but possess different mechanical strength, stiffness and brittleness similar to natural rocks; (2) randomly incorporating a certain amount of micro-scale pores and fractures to simulate the micro-scale defects in real rock masses; (3) the developed materials shall have a relatively low glass transition temperature than conventional polymer resins, which can be obtained through a series of modifications and alterations of the principal chemical constituents of printing materials (the printing materials can, therefore, exhibit good stress-frozen behaviours at temperatures close to room temperature); and (4) applying freezing treatment to enhance the brittleness of the 3D printable resin. Further attempts at material modification will be discussed in another paper.

## Methods

### Materials

The printable material of interest is a type of resinous material with the trade name VeroClear-RGD 810, which is a rigid, nearly colourless material exhibiting dimensional stability. It is an epoxy-based polymer with a polymerized density of 1.18–1.19 g/cm^3^ at room temperature. Additionally, it has a water absorption of 1.1–1.5%, Rockwell hardness of 73–76 scale M, and a heat distortion temperature (HDT) of approximately 45–50 °C. VeroClear was supplied by Stratasys Ltd., Israel. It is available for Objet Connex 3D printers.

### Specimen Preparation via 3D Printing

VeroClear is available for Objet Connex 3D printers and can be used to create a series of cylindrical and dog-bone-shaped specimens for mechanical property testing. The cylindrical and dog-bone shaped specimens were first created in AutoCAD®, exported in Stereo Lithographic (*STL*) format, and then imported into the Objet Studio program used to print each type of specimen. Note that the mechanical properties of photopolymers are dependent on UV exposure time and may be sensitive, to a certain extent, to variations in illumination^14, 51^. The storage time between printing and testing and the storage conditions of each specimen were controlled to be as uniform as possible.

**Figure 8:**
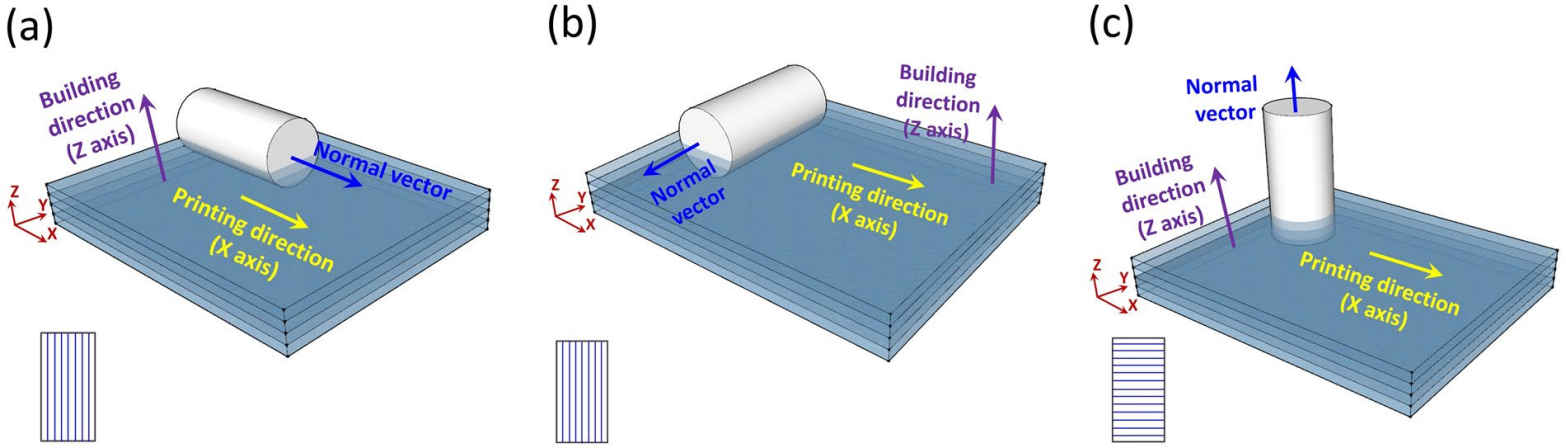
Schematic representation of 3D-printed samples with different build-up orientations, wherein the normal vector of the printed specimen is parallel to the (**a**) X-axis, (**b**) Y-axis and (**c**) Z-axis of the printing direction.

To investigate the effects of the build-up orientation on the mechanical properties of the printed samples, specimens of three types were prepared with the normal vector to the top surface parallel to the X-axis, Y-axis and Z-axis. Schematic diagrams are shown in Fig. 8. To eliminate the effect of the printing time (UV irradiation time) on the mechanical properties, three series of samples were printed separately. The symbols S-X, S-Y and S-Z indicate the building direction axis that is parallel to the normal vector of the sample.

### Heat treatment

Four heat treating temperatures, i.e., 60 °C, 90 °C, 120 °C and 150 °C, were taken into consideration for measuring the influence of temperature on the mechanical properties of the 3D-printed samples. S-Y was fixed as the build-up direction to eliminate the influence of different manners of building. To eliminate the thermal stress effect, we used relatively low heating and cooling rates of 6 °C per hour. All printed specimens were heat treated for 48 hours.

### Analysis of chemical composition

An FT-IR spectrometer (product model Spectrum 400) was employed to qualitatively analyse the molecular structure of VeroClear based on the absorption or transmittance of infrared radiation passing through the sample. A pyrolyser (model EGA/PY-3030D) and gas-chromatograph mass-spectrometer (product model QP2010-Ultra) were integrated and implemented to detect the organic components of the pyrolysis products. A scanning electron microscope (product model S4800) was used to produce images of the sample’s surface topography and composition. Additionally, an X-ray diffractometer (product model D8 advance) was used to identify the semi-crystalline and crystalline phases of the polymers and the molecular structures of the crystals.

### Determination of mechanical property

A digital servo-control universal testing machine with a load capacity of 100 kN was employed to exert continual compressive and tensile stresses on the printed specimens. All specimens were compressed and pulled at a controlled rate of 2 mm/min until failure. A fully digital servo hydraulic triaxial testing system was implemented to detect the compressive strengths of printed samples under triaxial loading conditions. The testing system can apply an axial load of up to 750 kN and a confining pressure of up to 80 MPa. The triaxial compressive strengths of printed samples under three different confining pressures, i.e., 5 MPa, 10 MPa and 15 MPa, were detected. The loading rate was 2 mm/min.

## Electronic supplementary material

Supplementary Information
