# Supplementary material for: The mechanical and photoelastic properties of 3D printable stress-visualized materials
Source: Sci Rep. 2017 Sep 7;7:10918. doi: 10.1038/s41598-017-11433-4 (PMC5589947; doi:10.1038/s41598-017-11433-4)
Supplement: Supplementary file 1 — Supplementary Information [file 41598_2017_11433_MOESM1_ESM.doc]

**Title: The mechanical and photoelastic properties of 3D printable** **stress-visualized materials**

**Authors:** Li Wang *^a^*, Yang Ju *^b, c *^*, Heping Xie *^d^*, Guowei Ma *^e^*, Lingtao Mao *^b^*, Kexin He *^a^*

*^a^ School of Mechanics and Civil Engineering, China University of Mining & Technology at Beijing, D11 Xueyuan RD, Beijing 100083, China*

*^b^ State Key Laboratory of Coal Resources and Safe Mining, China University of Mining and Technology at Beijing, D11 Xueyuan RD, Beijing 100083, China*

*^c^ State Key Laboratory for Geomechanics and Deep Underground Engineering, China University of Mining and Technology at Xuzhou, 1 University Ave, Xuzhou 221006, China*

*^d^ Key Laboratory of Energy Engineering Safety and Mechanics on Disasters, The Ministry of Education, Sichuan University, Chengdu 610065, China*

*^e^ School of Civil, Environmental and Mining Engineering, The University of Western Australia, Crawley, WA 6009, Australia*

***Corresponding author:**

Yang Ju, PhD

State Key Laboratory of Coal Resources & Safe Mining,

China University of Mining & Technology at Beijing,

D11 Xueyuan Road, Beijing 100083, China

Tel: +86 10 62331490; Fax: +86 10 62331253;

Email: [juy@cumtb.edu.cn](mailto:juy@cumtb.edu.cn) or [yju@icloud.com](mailto:yju@icloud.com)

**Supplementary Tables**

Supplementary Table ST-1 lists information about the methods and instruments adopted in this paper for the chemical composition analysis of the resinous printable material VeroClear.

**ST-1** Equipment used to analyse the chemical composition of VeroClear

| Method | Main function | Apparatus | Product Model | Key Specifications | Company |
| --- | --- | --- | --- | --- | --- |
| FT-IR | molecular structure identification | FT-IR spectrometer | Spectrum 400 | wavenumber 400-25000 cm^-1^ | [PerkinElmer](http://www.baidu.com/link?url=Akazu7eN-f3CjTppn-2WZwOHi9w-vgm2h1Y_12rk4beqzvp-OxZ83v93YWWczj-sjzWqJJ6n1JijA0llobM0na&wd=&eqid=81fb38430018bff2000000045760ff16) (USA) |
| SEM | microstructural observation | scanning electron microscope | S4800 | Magnification: x 800,000  resolution: 1.0 nm | Hitachi (Japan) |
| XRD | phase quantitative analysis | X-radial diffractometer | D8 advance | goniometer radius:  > 200 mm; 2θ: -10~168° | Bruker  (Germany) |
| PY | [organic material](https://en.wikipedia.org/wiki/Organic_matter) [decomposition](https://en.wikipedia.org/wiki/Decomposition) | pyrolyser | EGA/PY-3030D | pyrolytic temperature  10°C-1050°C, ±0.1 °C | Frontier (Japan) |
| GC-MS | organic component detection | gas-chromatograph mass-spectrometer | QP2010-Ultra | Max [scanning](javascript:void(0);) [speed](javascript:void(0);): 20,000 µm/sec | Frontier (Japan) |

Supplementary Table ST-2 presents the PY-GC/MS analysis results of the pyrolysis products of VeroClear at a 300℃.

**ST-2** Results of PY/GC-MS analysis of pyrolysis products of VeroClear at 300℃

| Peak | Time | Area (%) | Height (%) | SI | CAS | Structure |
| --- | --- | --- | --- | --- | --- | --- |
| 1 | 4.852 | 15.04 | 7.72 | 98 | 108-94-1 |  |
| 2 | 6.179 | 19.03 | 10.89 | 98 | 100-52-7 |  |
| 3 | 11.223 | 4.50 | 6.71 | 96 | 5117-12-4 |  |
| 4 | 12.906 | 51.20 | 60.28 | 96 | 5888-33-5 |  |
| 5 | 16.812 | 10.24 | 14.40 | 97 | 947-19-3 |  |

**Supplementary Figures**

Supplementary **Fig. SF-1** shows pictures of the 3D-printed cylinder and the dog-bone-shaped specimen and the corresponding detailed [geometrical](javascript:void(0);) information.


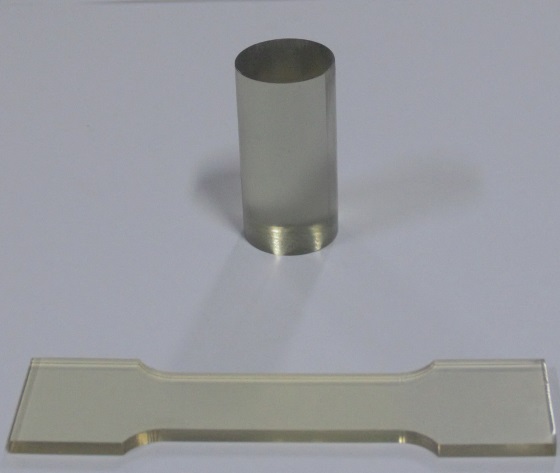
(***a***)
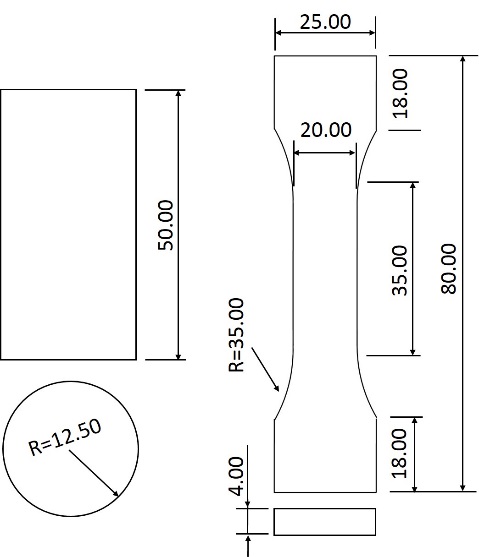
(***b***)

**SF-1** (***a***) 3D-printed specimen made of VeroClear, shown after removing the support structure, followed by smoothing and polishing; (***b***) specimen geometries with relevant dimensions in mm.

Supplementary **Fig. SF-2** depicts the heating procedure adopted to evaluate the influence of heat-treatment temperatures on the mechanical properties of the printable material VeroClear.


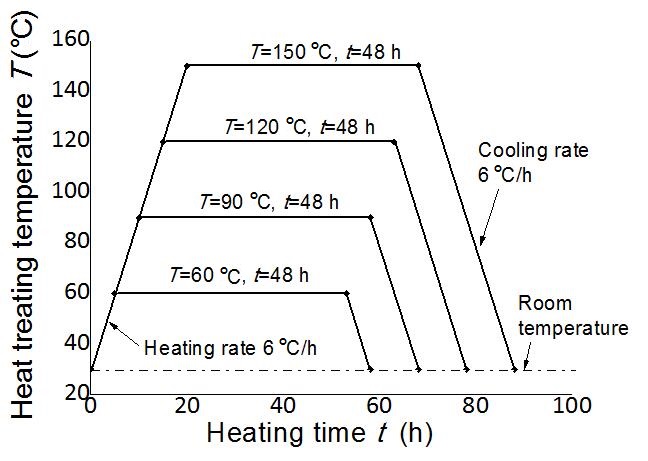


**SF-2**. Schematic diagram of the heating procedure.

Supplementary **Fig. SF-3** shows the testing setup for uniaxial compressive testing and direct tensile testing.


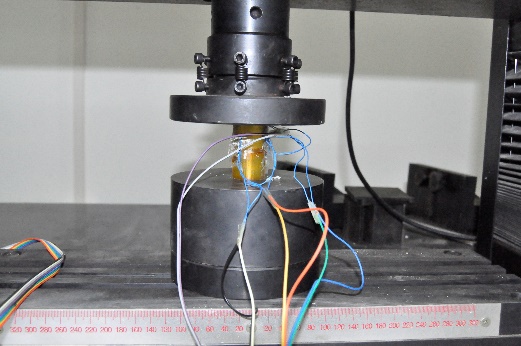
(***a***)
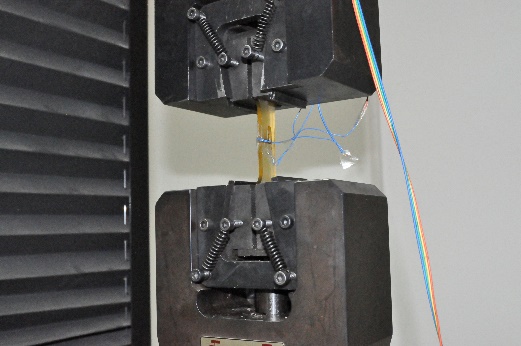
(***b***)

**SF-3** Setup for **(*a*)** Uniaxial compressive strength testing; **(*b*)** Direct tensile strength testing.

Supplementary **Fig. SF-4** shows the fully digital servo hydraulic triaxial testing system implemented to detect the compressive strengths of the printed samples under triaxial loading conditions.


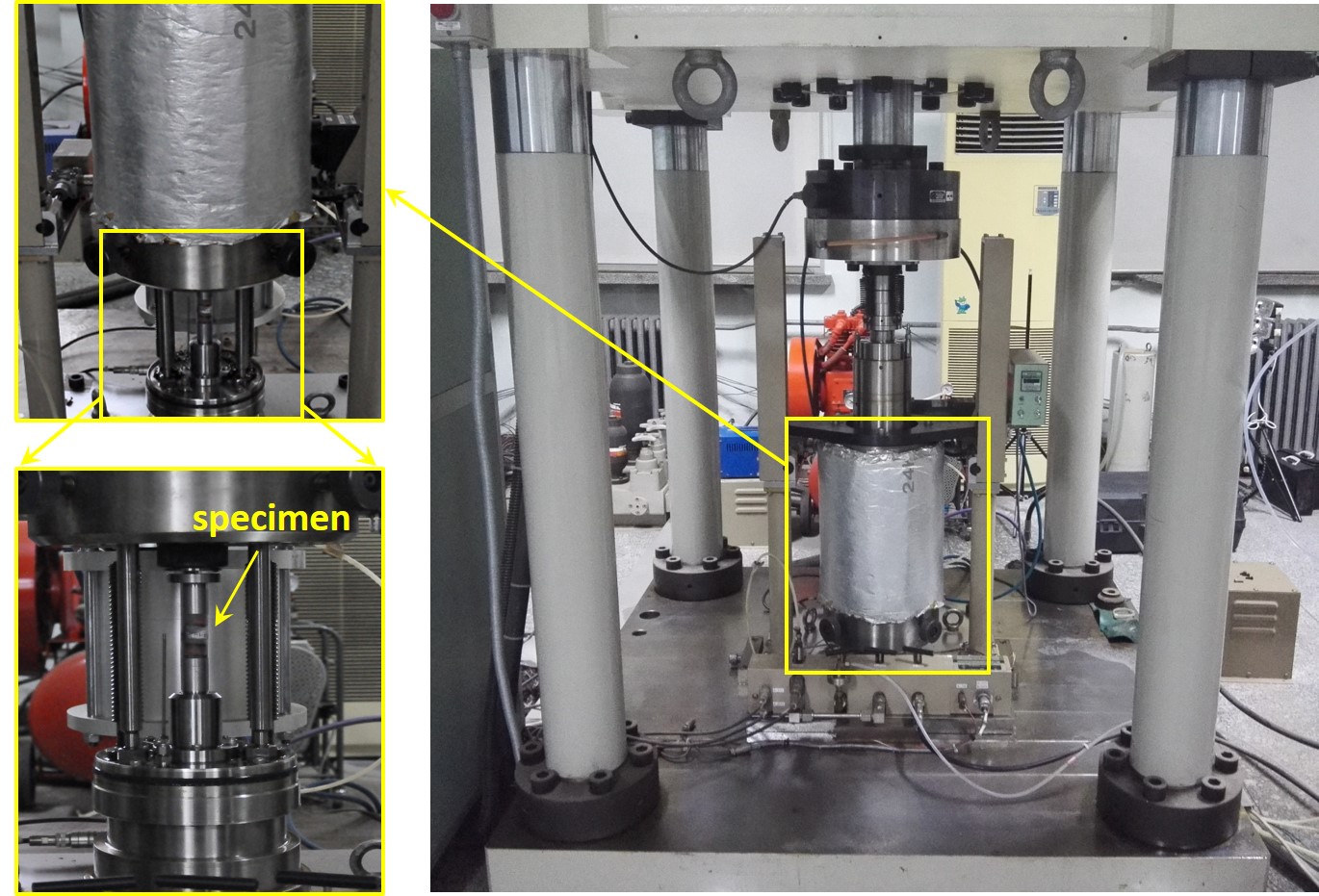


**SF-4** EHF-UG 500KN fully digital servo hydraulic triaxial testing system; the enlarged image shows the confining pressure chamber.

Supplementary **Fig. SF-5** presents the uniaxial compressive strength and elastic modulus data of specimens printed in different build-up orientations.


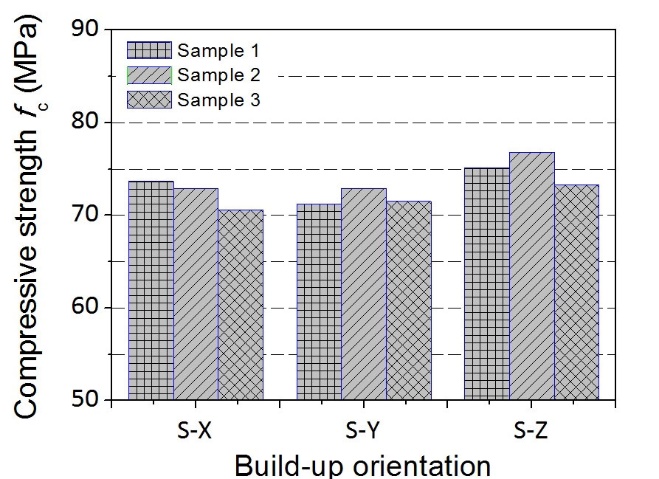
(***a***)
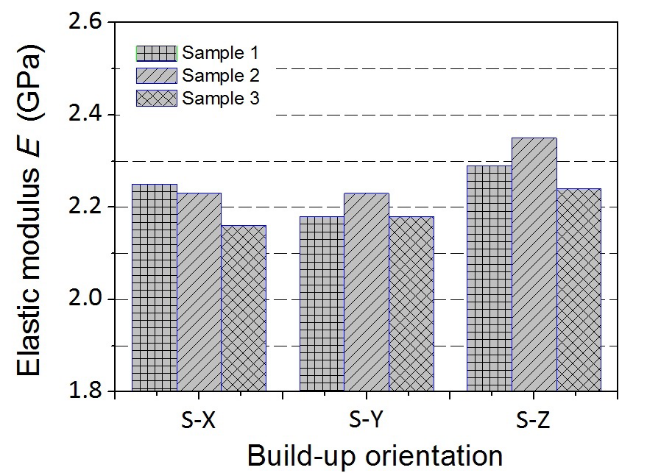
(***b***)

**SF-5** Comparison of (***a***) UCS and (***b***) elastic modulus of cylinders with built in different manners. Three samples were tested for each case.

Supplementary Fig. SF-6 depicts the hot-light curve, which characterizes the relationship between deformation and temperature, of the material VeroClear.


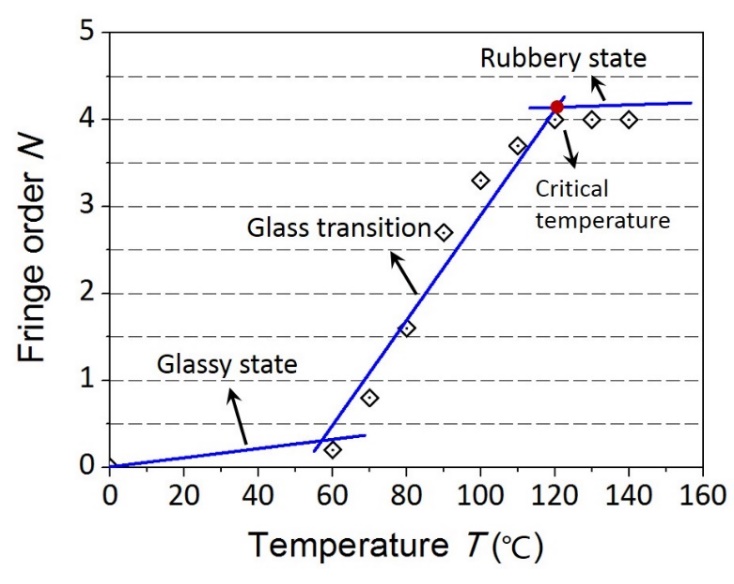


**SF-6** Hot-light curve of VeroClear
